# Risk stratification for failure of conservative treatment in a cohort of 270 diametaphyseal radius fractures

**DOI:** 10.1007/s00402-025-05929-2

**Published:** 2025-05-30

**Authors:** Ricardo Beck, Susann Marie Beck, Eric Waltersbacher, Alexandra Wilke, Matthias Kuhn, Guido Fitze, Philipp Schwerk, Jurek Schultz, Christoph von Schrottenberg

**Affiliations:** 1 Department of Pediatric Surgery, Faculty of Medicine and University Hospital Carl Gustav Carus, TUD Dresden University of Technology, Fetscherstraße, 74, 01307 , Dresden, Germany; 2 Institute for Medical Informatics and Biometry, Faculty of Medicine, Technical University Carl Gustav Carus, Dresden, Germany

**Keywords:** Diametaphyseal radius fracture, Distal radius fracture, Conservative fracture management, Pediatric forearm fracture

## Abstract

**Background:**

Diametaphyseal radius fractures (DMRF) in children pose a challenge to pediatric traumatologists. Numerous techniques to address the problems of conventional osteosynthesis have been published. Still, immobilization in a cast is the treatment of choice in most cases. The aim of this study was to assess risk factors for the failure of conservative treatment in DMRFs.

**Methods:**

This is a single-center, retrospective study of 270 patients with conservatively treated DMRFs. Demographic data and radiologic fracture characteristics were assessed. Univariate and multivariate regression analyses were performed to identify risk factors for secondary dislocation, refracture, or secondary osteosynthesis. Significant variables were included in a risk prediction model.

**Results:**

Secondary dislocation, refracture, and secondary osteosynthesis occurred in 10.0%, 5.6%, and 11.0%, respectively. Increasing angles of the fracture line, severe angulation, and greenstick fractures were identified to significantly predict either one of these complications. Surprisingly, proximal DMRFs with an increased forearm fracture index were less likely to dislocate secondarily.

**Conclusions:**

Conservatively treated DMRFs with severe angulation, tilted fracture lines, and greenstick fractures are more likely to suffer complications. Hence, these fractures should be stabilized with osteosynthesis primarily. The introduced risk prediction tools should be validated prospectively.

**Supplementary Information:**

The online version contains supplementary material available at 10.1007/s00402-025-05929-2.

## Introduction

Distal radius fractures are very common in children [17, 18, 23]. Due to the great remodelling capacity, most cases can be treated conservatively [22]. Closed reduction and immobilization alone can be sufficient even when the physes are closed [30]. This eliminates the risk of osteosyntheses-related infections that has been reported in adults, but might be less relevant in children [8, 16, 28, 32].

In contrast to most distal radius fractures, diametaphyseal radius fractures (DMRFs) present a relevant challenge to pediatric traumatologists [21]. Numerous surgical techniques have been published, including transepiphyseal intramedullary Kirschner-wire osteosynthesis (TEPIK), antegrade nailing of the radius as well as modified retrograde intramedullary nailing techniques and plate osteosynthesis [3, 10, 11, 20, 25, 29]. Complications such as secondary dislocation after surgical treatment are mostly due to the use of inappropriate surgical techniques [2, 24]. While determining the best surgical technique to stabilize DMRFs is controversially discussed, so is the mere definition of the diametaphyseal junction zone (DMJZ) [36, 38]. Lieber et al. defined the DMJZ as the area of the radius within the square of the combined width of radius and ulna growth plate minus the square of the width of the radius growth plate alone [24]. In 2025, another definition of the DMJZ, which extends slightly more proximally, was introduced using the forearm fracture index (FFI). With the FFI, the exact localization of DMRFs within the DMJZ can be pinpointed. The FFI is calculated by the ratio of the fracture’s true distance to the growth plate assessed in a lateral radiograph over the width of the radius growth plate assessed in an anterior-posterior (a.p.) radiograph [37]. The higher the FFI, the more proximal the fracture is.

The remodelling capacity of the diametaphyseal junction zone is unclear and there is no consensus on the degree of dislocation that can be tolerated, nor have any other variables been defined to clarify the indication for surgery in DMRFs. The rate and causes of complications in conservatively treated DMRFs have not been sufficiently investigated [24]. In this study, we analyzed fracture characteristics, clinical courses, and complications of 270 conservatively treated DMRFs. We hypothesize that a greater FFI is associated with a higher risk of secondary dislocation, as the diameter of the bone decreases, the more proximal the fracture lies. This leads to a reduced contact surface of the two fragments, which may facilitate secondary dislocation.

## Materials and methods

### Data acquisition

We retrospectively analyzed all forearm fractures in patients 16 years or younger treated at our institution from 2010 to 2020. The study was approved by our local ethics committee (EK 433102016). Data was retrieved using ICD-Codes S52.0 – S52.9. Duplicates were eliminated. Further exclusion criteria were falsely coded fractures, solitary fractures of the ulna, pathological fractures, buckle fractures, fractures lost to follow-up and closed growth plates in the initial radiograph. A patient flow diagram is provided in the Supplementary Material (Figure S1) [6]. Three types of complications were studied: secondary dislocation, refracture, and secondary osteosynthesis. Secondary dislocation was defined as a dislocation of the fracture within 4 weeks after the initial trauma requiring reduction. Refracture was defined as a fracture of the same bone in the same localization after consolidation but within 6 months after the initial trauma. Secondary osteosynthesis was registered if a patient underwent unplanned surgery and had the fracture stabilized with an osteosynthesis. To identify risk factors that could potentially influence the incidence of complications in conservatively treated DMRFs, the following fracture characteristics in initial a.p. and lateral radiographs were assessed: greenstick fracture, concomitant ulna fracture, shortened fracture, fracture angulation in degree, translation of the fragment as percentage of the shaft’s width at the level of the fracture, angle of the fracture line in degree. Additionally, the FFI was assessed, and DMRFs(+) were identified when the fracture had an FFI between 1 and 2, but was too proximal to be considered a DMRF as defined by Lieber et al. [25, 37].

### Statistics

**Fig. 1:**
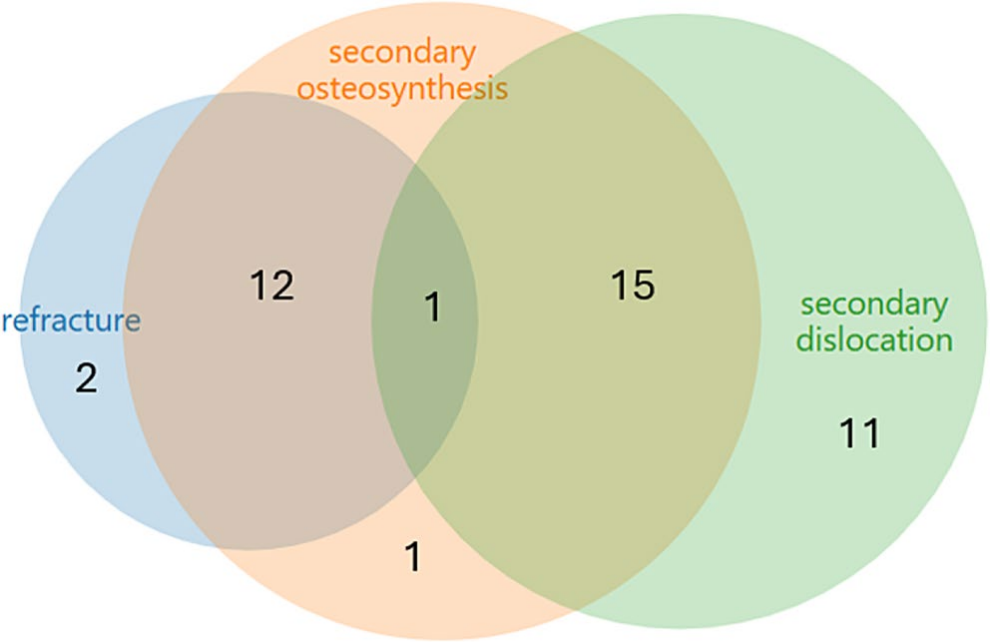
Area-proportional Venn diagram of complications during follow-up in 270 pediatric, conservatively managed diametaphyseal radius fractures

Demographic and clinical data are displayed as means with standard deviation. If non-normally distributed, data are displayed as medians with interquartile range. To identify risk factors for the occurrence of complications, we performed univariate and then multiple logistic regression analysis. Predictors with a *p* < 0.1 in the univariate and in the multiple regression were retained. For the regression, we log-transformed the angle of fracture line variables to remedy the right skew. We used the Akaike information criterion (AIC) to further select predictors within the multiple regression model. As the low incidence of complications makes spurious findings likely, we fitted all logistic regression models using Firth’s correction for bias reduction [19]. Moreover, we used AIC with a strong penalty parameter k, depending on the number of observed complications, namely k = 3 for secondary dislocation and secondary osteosynthesis and k = 5 for refracture. Standardized odds ratios (std. OR) are given in order to make results more comparable [13]. To yield a simple score model, we categorized continuous predictors according to quantiles of their distribution in the data. Then, for all predictors, we assigned integer score points for each category that most closely resembled the estimated effect in log-odds for going from the midpoint of the lowest category to the midpoint of each category. Predictive performance of each model was analyzed via ROC analysis. For statistical analysis, we used the software environment R. The Venn Diagram in Fig. 1 was produced using the Dataset Overlap and Venn Diagram web tool from BioInfoRx (https://bioinforx.com/apps/venn_overlap.php [accessed 17.02.2025]).

## Results

### Demographic data and fracture characteristics

270 DMRFs were treated conservatively with immobilization in an upper arm cast. Demographic data, fracture characteristics, and complications are displayed in Table 1. Secondary dislocation occurred in 27 (10%), and refractures in 15 (5.6%) patients. Secondary osteosynthesis was necessary in 29 (11%) patients. The co-occurrence of the different complications is displayed in a Venn diagram in Fig. 1.

**Table 1:** Demographic data, fracture characteristics and complications of 270 patients with conservatively treated diametaphyseal radius fractures. °, angle in degree; DMRF(+), fractures of the distal radius that were defined as diametaphyseal with a forearm fracture index between 1 and 2 but would be considered diaphyseal according to Lieber et al.

	Cohort
	(*n* = 270)
Age (years)	8 (5,10)
Male	189 (70%)
Greenstick fractures	136 (50%)
Concomitant ulna fracture	191 (71%)
Shortened fracture	19 (7.3%)
DMRF(+)	10 (3.7%)
Forearm fracture index	1.34 ± 0.28
Angulation in the a.p. radiograph (°)	5 (0,10)
Angulation in the lateral radiograph (°)	18 (14,24)
Angle of fracture line in the a.p. radiograph (°)	7 (0,15)
Angle of fracture line in the lateral radiograph (°)	13 (0,24)
Translation in the a.p. radiograph	55 (21%)
Translation in the lateral radiograph	39 (15%)
Secondary dislocation	27 (10%)
Refracture	15 (5.6%)
Secondary osteosynthesis	29 (11%)

### Defining risk factors for complications in conservatively treated DMRFs

In univariate analysis, a more distal fracture with smaller FFI, increased angulation in the lateral radiograph, increased angle of the fracture line in both a.p. and lateral radiographs, and greater translation of the fracture in the lateral radiograph were all significantly associated with secondary dislocation. In contrast, refractures were associated with being DMRF(+) and thus more proximal. Additionally, greenstick fractures and fractures with an increased angle of the fracture line in both a.p. and lateral radiographs were also associated with refractures. Secondary osteosynthesis was associated with older age, DMRF(+), and an increased angle of the fracture line in both a.p. and lateral radiographs. Results of univariate analysis are displayed in Table 2.

**Table 2:** Univariate logistic regression of variables associated with the incidence of complications (secondary dislocation, refracture and secondary osteosynthesis). P-values are displayed; °, angle in degree; DMRF(+) are fractures that were defined as diametaphyseal with a forearm fracture index between 1 and 2, but diaphyseal according to Lieber et al.’s definition

	Secondary dislocation	Refracture	Secondary osteosynthesis
	(*n* = 27)	(*n* = 15)	(*n* = 29)
Age	0.26	0.20	**0.075**
Concomitant Ulna fracture	0.45	0.32	0.48
Shortened fracture	0.70	0.79	0.79
DMRF(+)	0.72	**0.036**	**0.046**
Greenstick	0.81	**0.007**	0.59
Forearm fracture index	**0.066**	0.95	0.55
Angulation in the a.p. radiograph	0.37	0.98	0.87
Angulation in the lateral radiograph	**0.056**	0.41	0.40
Angle of fracture line in the a.p. radiograph	**< 0.001**	**0.007**	**0.001**
Angle of fracture line in the lateral radiograph	**0.023**	**0.015**	**0.01**
Translation in the a.p. radiograph	0.15	0.13	0.80
Translation in the lateral radiograph	**0.013**	0.22	0.50

**Fig. 2:**
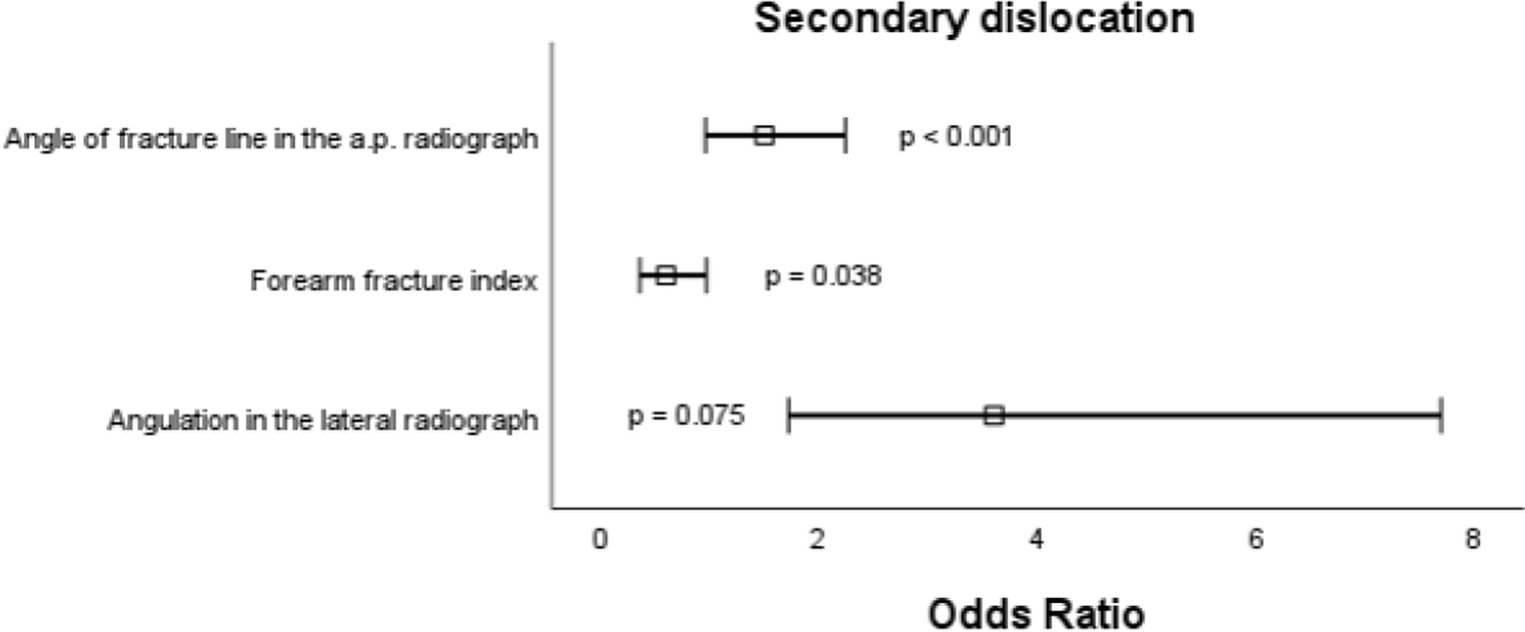
Forest plot of significant risk factors for secondary dislocation in conservatively treated diametaphyseal radius fractures. Boxes represent the standardized odds ratio und whiskers represent the 95% confidence interval; a.p., anterior-posterior

**Fig. 3:**
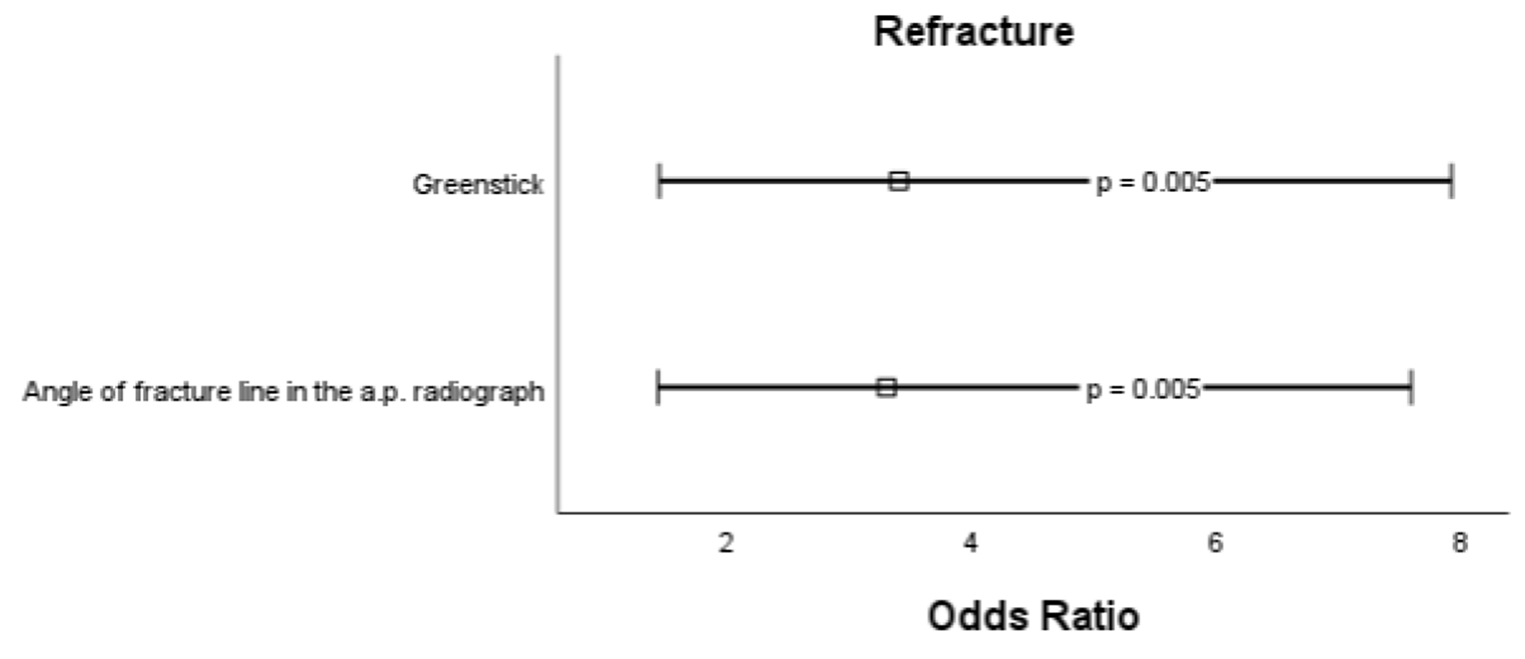
Forest plot of significant risk factors for refracture in conservatively treated diametaphyseal radius fractures. Boxes represent the standardized odds ratio und whiskers represent the 95% confidence interval; a.p., anterior-posterior

**Fig. 4:**
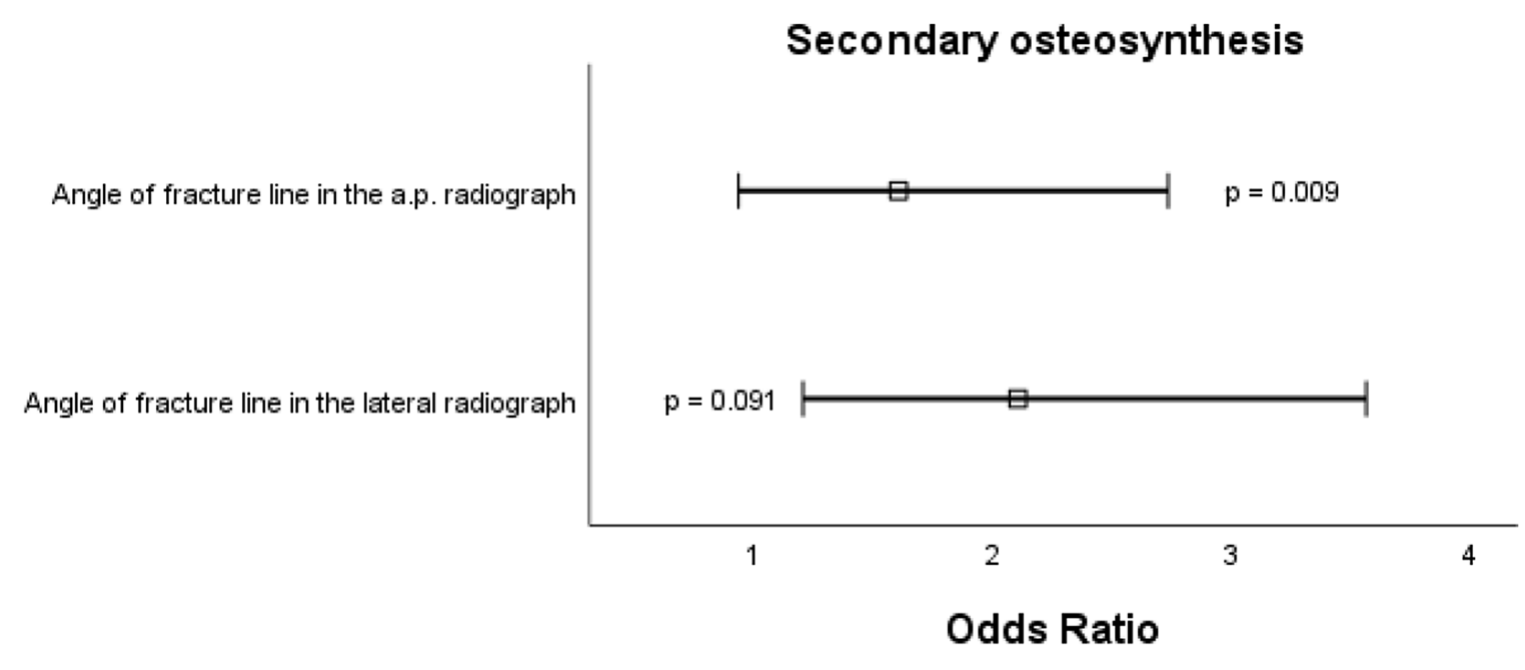
Forest plot of significant risk factors for secondary osteosynthesis in conservatively treated diametaphyseal radius fractures. Boxes represent the standardized odds ratio und whiskers represent the 95% confidence interval; a.p., anterior-posterior

Next, bias-reduced multiple logistic regression was performed to identify independent risk factors for each complication [5]. For secondary dislocation, the following variables persisted as risk factors: angle of fracture line in the a.p. radiograph (std. OR 3.63, 95% CI 1.72–7.69, *p* < 0.001), angulation in the lateral radiograph (std. OR 1.47, 95% CI 0.96–2.24, *p* = 0.075). Intriguingly, a greater FFI reduced the risk for secondary dislocation (std. OR 0.59, 95% CI 0.36–0.97, *p* = 0.038). For refracture, the following variables persisted as risk factors: increased angle of fracture line in the a.p. radiograph (std. OR 3.29, 95% CI 1.43–7.59, *p* = 0.005) and greenstick fracture (std. OR 3.38, 95% CI 1.44–7.92, *p* = 0.005). For secondary osteosynthesis, the following variables persisted as risk factors: angle of fracture line in the a.p. radiograph (std. OR 2.06, 95% CI 1.20–3.56, *p* = 0.009) and angle of fracture line in the lateral radiograph (std. OR 1.59, 95% CI 0.93–2.73, *p* = 0.091). Odds ratios of all risk factors including p-values are displayed as forest plots in Figs. 2, 3 and 4.

### Risk prediction scores for complications in the conservative management of DMRFs

Risk prediction scores for the occurrence of each of the three complications were designed and are displayed in Tables 3, 4 and 5. The area under the curve (AUC) of the three risk prediction models was 0.80, 0.84 and 0.73, respectively. Figure S2 in the Supplementary Material visualizes the ROC analysis of the risk prediction model for secondary dislocation, exemplarily.

#### Risk prediction score for secondary dislocation

**Table 3:** Risk prediction score including all significant risk factors for secondary dislocation in conservatively managed diametaphyseal radius fractures. A.p., anterior-posterior

Forearm fracture index	Points	Angulation in the lateral radiograph	Points	Angle of fracture line in the a.*p*. radiograph	Points	Sum	Risk
> 1.50	0	0–20°	0	0°	0	**0**	**0.2%**
1.21–1.50	1	21–40°	1	1–10°	2	**1**	**0.5%**
1.0–1.20	2	> 40°	2	10–20°	3	**2**	**1.1%**
				> 20°	4	**3**	**2.6%**
						**4**	**6.0%**
						**5**	**13.2%**
						**6**	**26.8%**
						**7**	**46.7%**
						**8**	**67.8%**

**Fig. 5:**
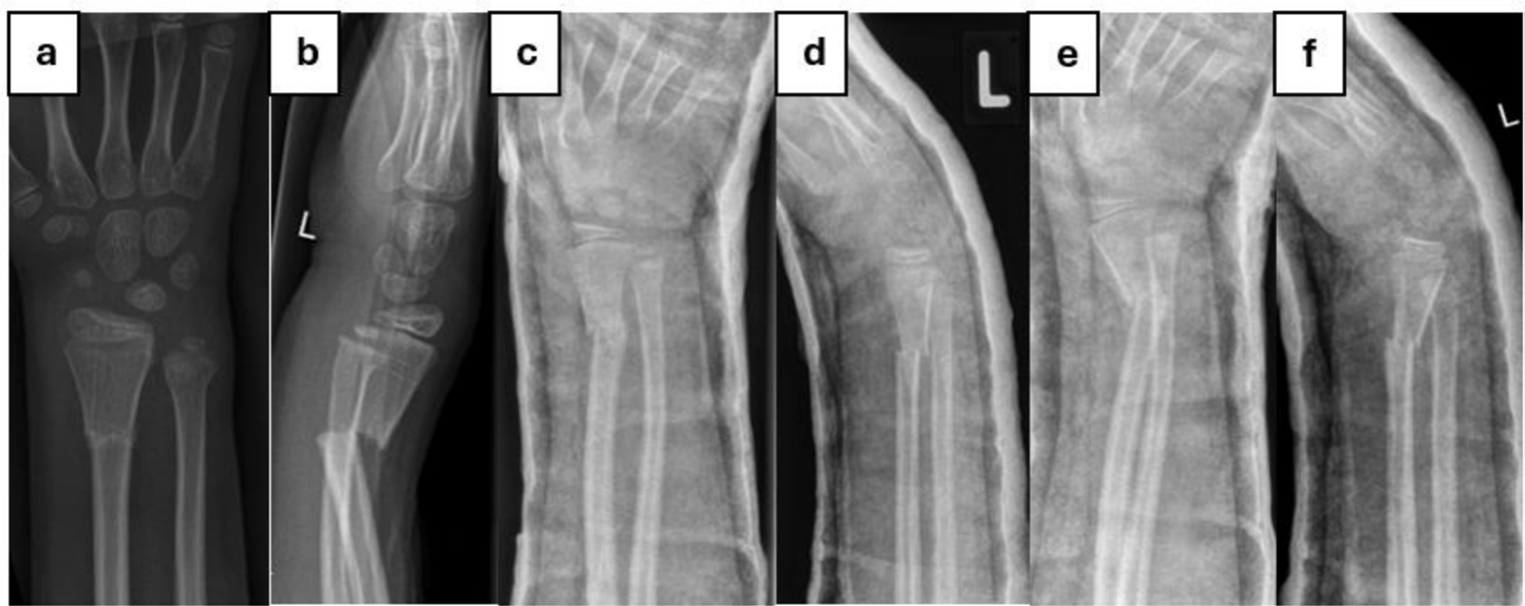
**a – b**, anterior-posterior and lateral radiographs of a dislocated diametaphyseal radius fracture in a 6-year-old boy at the day of the accident (day 0). The forearm fracture index is 1.2, the angulation in the lateral radiograph is 30° and the angle of the fracture line in the a.p. radiograph is approximately 2°. Hence, the risk for secondary dislocation according to the proposed risk prediction score in Table 3 is 13.2%. Initially, the fracture was managed conservatively; **c – d**, radiologic imaging shows an acceptable retention of the reduced fracture on day 7; **e – f**, radiologic imaging shows a considerable secondary dislocation of the fracture, particularly in the anterior-posterior plane on day 16

Fig. 5 displays a DMRF that was initially managed conservatively and dislocated secondarily. Applying the risk prediction score presented in Table 3 (FFI of 1.2 = 2 points, angulation of 30° in the lateral radiograph = 1 point, angle of the fracture line in the a.p. radiograph of approximately 2° = 2 points, in sum 5 points), this particular DMRF had a risk for secondary dislocation of 13.2%.

#### Risk prediction score for refractures

**Table 4:** Risk prediction score including all risk factors for refracture in conservatively managed diametaphyseal radius fractur. A.p., anterior-posterior

Greenstick fracture	Points	Angle of fracture line in the a.*p*. radiograph	Points	Sum	Risk
No	0	0°	0	**0**	**0.1%**
Yes	3	1–10°	2	**1**	**0.3%**
		11–20°	3	**2**	**0.7%**
		> 20°	4	**3**	**1.6%**
				**4**	**3.5%**
				**5**	**7.6%**
				**6**	**15.7%**
				**7**	**29.5%**

**Fig. 6:**
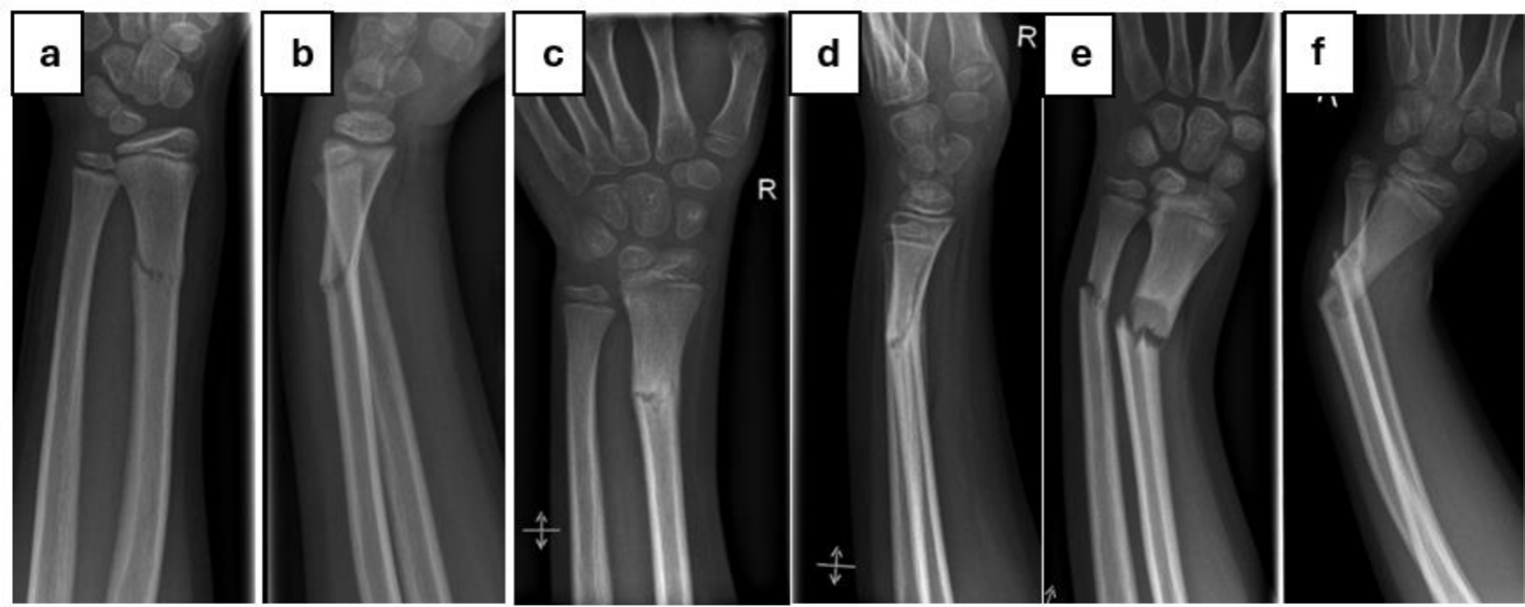
**a – b**, anterior-posterior and lateral radiographs of a diametaphyseal radius greenstick fracture in a 10-year-old boy at the day of the accident (day 0). The angle of the fracture line in the a.p. radiograph is approximately 25°. Hence, the risk for refracture according to the proposed risk prediction score in Table 4 is 29.5%; **c – d**, radiologic imaging shows consolidation in an acceptable position on day 33; **e – f**, radiologic imaging shows a refracture on day 147

Figure 6 displays a greenstick DMRF that was initially managed conservatively and refractured during follow-up. Applying the risk prediction score presented in Table 4 (greenstick fracture = 3 points, angle of the fracture line in the a.p. radiograph of 25° = 4 points, in sum 7 points), this particular DMRF had a risk for refracture of 29.5%.

#### Risk prediction score for secondary osteosynthesis

**Table 5:** Risk prediction score including all risk factors for secondary osteosynthesis in conservatively managed diametaphyseal radius fractures. A.p., anterior-posterior

Angle of fracture line in the lateral radiograph	Points	Angle of fracture line in the a.*p*. radiograph	Points	Sum	Risk
0	0	0	0	**0**	**1.8%**
> 0°	1	1–10°	1	**1**	**4.3%**
		> 10°	2	**2**	**10.4%**
				**3**	**22.8%**

## Discussion

Conservative treatment of distal radius fractures in children is routinely performed with relatively clear benchmarks regarding the dislocations that can be tolerated due to the great remodelling capacity of the distal radius [12]. In contrast, the unknown potential for spontaneous alignment and the instability of DMRFs encourage surgeons to perform osteosynthesis without having clear guidance on which fractures could potentially be managed conservatively.

We investigated 270 DMRFs that were managed conservatively with immobilization in an upper arm cast, to identify risk factors that could facilitate failure of conservative treatment. If either secondary dislocation, refracture or secondary osteosynthesis occurred during follow-up, this was considered failure of conservative treatment. At least one of these complications occurred in 42 of the 270 patients, adding up to an overall complication rate of 15.6%. While secondary osteosynthesis was the consequence of either a secondarily dislocated fracture or a refracture during follow-up, 11/27 (41%) patients with secondary dislocation could still be managed conservatively with another reduction or wedging of the cast in an outpatient setting without anesthesia or osteosynthesis. 13/15 (87%) patients who suffered refractures ultimately were operated on and had the radius stabilized with osteosynthesis.

### Risk factors for secondary dislocation in conservatively managed DMRFs

For conservatively managed metaphyseal radius fractures, the following independent risk factors for secondary dislocation have been identified: complete dislocation of the fragment, combined complete fracture of radius and ulna and non-anatomic reduction of a dislocated fracture [14, 15, 33, 34]. However, for diametaphyseal fractures, no such risk factors have been reported in the literature yet.

As our data show, the risk for secondary dislocation of conservatively treated DMRFs correlates with the angle of the fracture line in the a.p. radiograph. It is easy to imagine that a tilted fracture surface facilitates a shift of the distal fragment whereas a horizontal fracture line will entail a more stable situation. Additionally, angulation of the distal fragment in the lateral radiograph was identified as a risk factor for secondary dislocation. Fractures were reduced in the emergency department (ED) when displaced, but it is comprehensible that initially more angulated fragments may tend to relapse.

Interestingly, the FFI turned out to be inversely correlated with the occurrence of secondary dislocations. This is surprising, as an increased FFI describes a more proximal localization of the fracture within the DMJZ. This goes along with a reduced contact surface of the two fragments as the bone diameter decreases versus the proximal zone of the DMJZ. While we expected that this would lead to an increased instability of the fracture, this hypothesis was not corroborated by our data. A certain bias cannot be excluded as our institution’s standard operating procedure calls for more proximal fractures to be stabilized with osteosynthesis more liberally. Consequently, there are only three fractures in our cohort with an FFI ≥ 1.8 that were managed conservatively. Thus, the small analyzed group of conservatively managed DMRFs with a high FFI in this study may represent a subgroup that was estimated to be particularly stable despite being so proximal. Another reason may be that longer fragments are supported by a longer part of the cast, whereas smaller fragments cannot be immobilized as effectively.

### Risk factors for refractures in conservatively managed DMRFs

While greenstick fractures of the forearm shaft are well investigated to be prone to refracture, this has not been demonstrated for DMRFs [4, 31]. In our cohort, 50.4% were greenstick fractures. Our data support that greenstick fracture is a highly significant risk factor for the occurrence of refractures in DMRFs with an OR of 3.4 (*p* = 0.005). Petnehazy et al. report a refracture rate of 14.5% in conservatively managed pediatric diaphyseal greenstick fractures of the forearm [27]. Comparably, the rate of refractures of conservatively managed greenstick fractures of the DMJZ was 10.3% in our cohort. Whether completing the fracture at the time of casting can prevent refractures during follow-up, is controversially discussed. Petnehazy et al. conclude that in conservatively managed diaphyseal fractures, this measure does reduce the rate of refractures during follow-up, while fractures that are stabilized with elastic stable intramedullary nails (ESIN) do not profit from being completed. In contrast, Schmuck et al. did not demonstrate any effect of this measure on the rate of refractures, but were able to show that residual angulation after consolidation could be reduced if the fracture had been completed at the time of casting [27, 31]. The second risk factor for refractures is an increasing angle of the fracture line in the a.p. radiograph. This may be connected to the increased risk of secondary dislocation, which may cause refracture after untypical consolidation of the fracture. Another cause may be that the callus in these tilted fractures is not arranged parallelly on both sides of the fracture but with a slight longitudinal offset to each other [7]. This may lead to insufficient stability of the callus and enhance refractures.

### Risk factors for secondary osteosynthesis in conservatively managed DMRFs

The angle of the fracture line in both a.p. and lateral radiographs were identified as risk factors for secondary osteosynthesis during follow-up. As outlined in 4.1. and 4.2., the angle of the fracture line in the a.p. radiograph significantly predicts the rate of secondary dislocations and refractures in DMRFs, which in many cases lead to a change of the therapeutic regime, i.e. reduction and osteosynthesis. Even though the angle of the fracture line in the lateral radiograph did not persist as a risk factor for secondary dislocation or refracture in the multiple logistic regression analysis, it was still significantly associated with both complications in the univariate regression analysis. As the angle of the fracture line in the lateral radiograph persisted as an independent risk factor for secondary osteosynthesis, this suggests that it may have similar effects on fracture stability as the angle of the fracture line in the a.p. radiograph.

### Risk prediction model for the conservative treatment of DMRFs

To reduce complications, patients suitable for conservative treatment should be selected according to evidence-based guidelines. High-risk fractures may benefit from surgical treatment in order to avoid complications. Consequently, risk prediction models were developed for each complication. AUC values ranging from 0.84 to 0.73 indicate good model performance.

The threshold to surgery may vary for each clinician who certainly must consider patients individually. Yet, with ever improving surgical techniques for stabilizing DMRFs, and an extremely low complication rate associated, conservative treatment is only justified if the risk for complication is minimal [3, 10, 25]. To keep the risk for secondary dislocation of a conservatively treated DMRFs below 5%, all patients with a score ≥ 4 in the risk prediction model (Table 3) should be considered for osteosynthesis. This includes all DMRFs with an angle of the fracture line in the a.p. radiograph of > 20°. Severe angulations of the distal fragment in the lateral radiograph (> 40°) combined with a tilted fracture line also lead the way to osteosynthesis, as the risk for secondary dislocation after reduction and immobilization in a cast will be eminent (> 6%).

Similarly, the risk of refracture should be accounted for when considering conservative treatment. The risk of < 5% may be considered a reasonable threshold. Hence, to avoid refractures, all DMRFs with a score ≥ 5 in the risk prediction model (Table 4) should be considered for osteosynthesis. This includes all greenstick fractures with any tilt of the fracture line in the a.p. radiograph.

Secondary osteosynthesis in conservatively managed patients causes considerable morbidity. Hence, they need to be avoided whenever possible. DMRFs with a score of ≥ 2 in the risk prediction model (Table 5) should therefore be considered for osteosynthesis primarily. Interestingly, this includes all patients with any tilt of the fracture line in both a.p. and lateral radiographs and all patients with a tilt of the fracture line of > 10° in the a.p. radiograph.

### Limitations

We did not consider delayed union or malunion as a possible complication in our analysis. In literature, the reported rate of delayed union in pediatric forearm fractures is very low (< 1%) and predominantly affects the ulna shaft [1, 9, 26]. Another limitation of this study is that cast quality was not assessed [35]. Furthermore, neurologic and motor-function outcomes were not analyzed. Instead, this study focused on the mechanical aspects of fracture stability. While this study provides a robust risk prediction tool for secondary dislocation, refracture and secondary osteosynthesis in conservatively managed DMRFs, external validation of these risk prediction scores still needs to be performed.

## Conclusion

With 270 patients, this is the largest, retrospective study assessing risk factors for the failure of conservative treatment of DMRFs in children and adolescents. We introduced an empirical-based risk prediction model for each of these complications: secondary dislocation, refracture and secondary osteosynthesis. DMRFs with a tilted fracture line in both radiographic planes, greenstick fractures with a tilted fracture line in either one of the two radiographic planes and fractures with a severe angulation of the distal fragment, should be considered for surgery. In the future, prospective, randomized controlled trials are needed to determine the utility of our proposed risk prediction scores.

## Electronic supplementary material

Below is the link to the electronic supplementary material.

Supplementary Material 1

## Data Availability

Datasets are available upon request to the corresponding author.
