# Supplementary material for: Risk stratification for failure of conservative treatment in a cohort of 270 diametaphyseal radius fractures
Source: Arch Orthop Trauma Surg. 2025 May 30;145(1):325. doi: 10.1007/s00402-025-05929-2 (PMC12125109; doi:10.1007/s00402-025-05929-2)
Supplement: Supplementary file 1 — Supplementary Material 1 [file 402_2025_5929_MOESM1_ESM.docx]

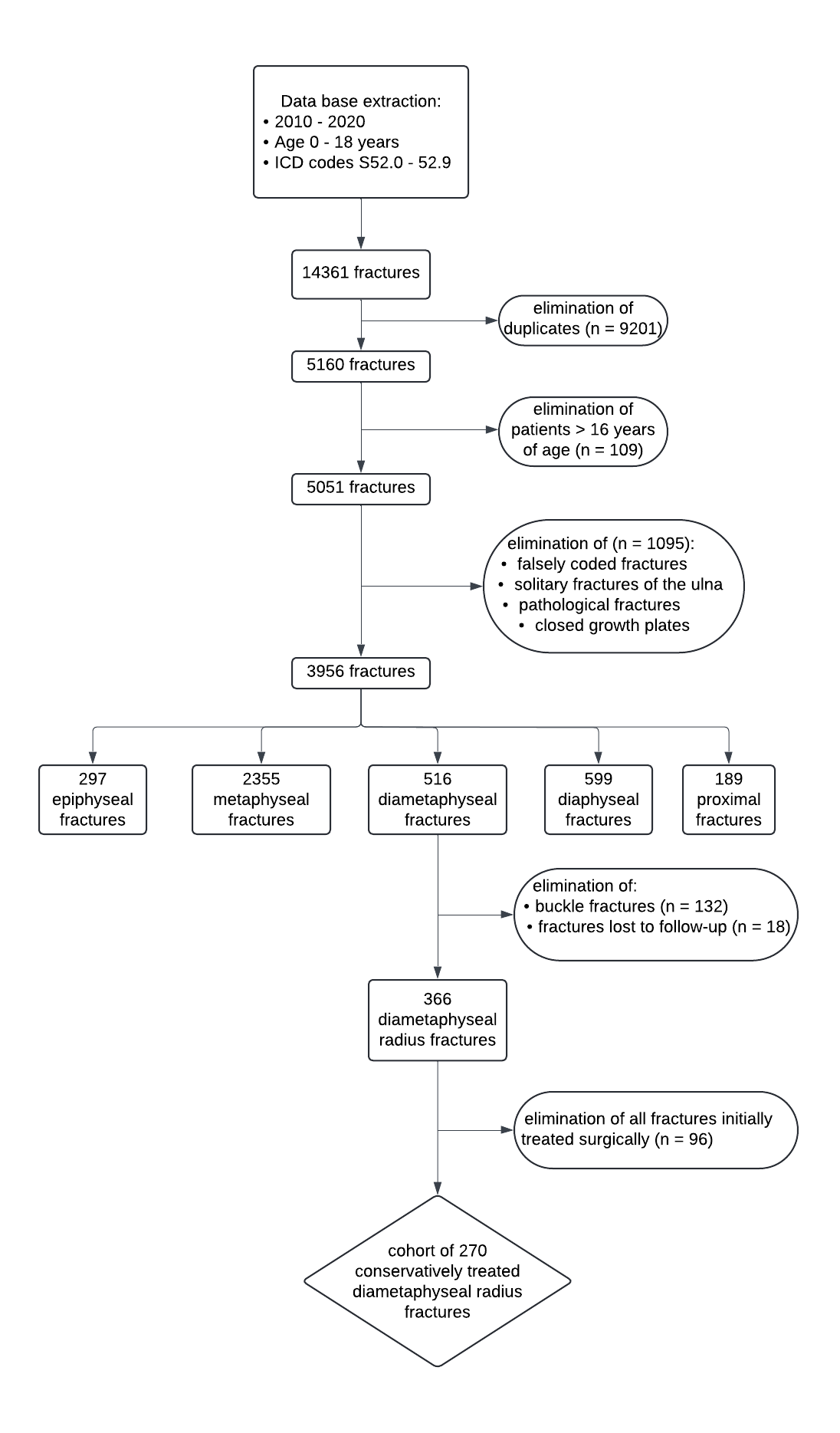
Supplementary Material

**Figure S1**. Flow diagram of patient acquisition.


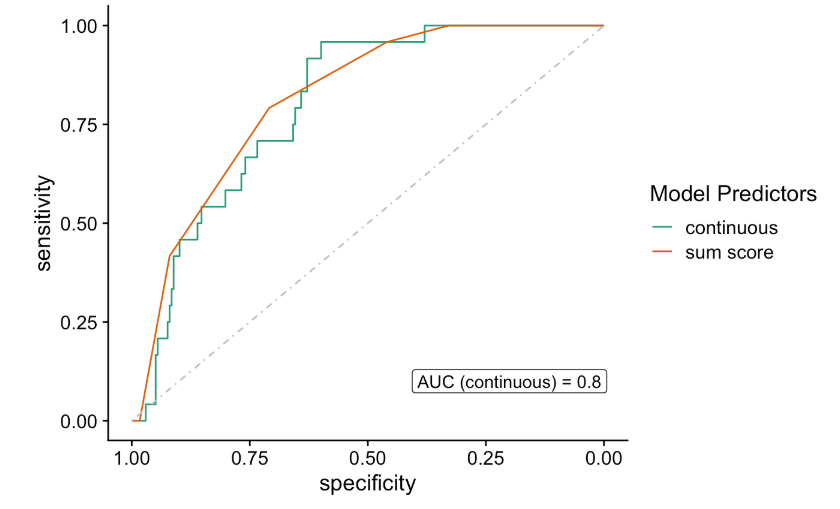


**Figure S2**. ROC analysis to determine predictive performance of the risk prediction score model for secondary dislocation. The area under the curve (AUC) is 0.8.
